# Supplementary material for: Lymphatic filariasis endgame strategies: Using GEOFIL to model mass drug administration and targeted surveillance and treatment strategies in American Samoa
Source: PLoS Negl Trop Dis. 2023 May 18;17(5):e0011347. doi: 10.1371/journal.pntd.0011347 (PMC10231811; doi:10.1371/journal.pntd.0011347)
Supplement: S1 Text — Description of the synthetic population model of American Samoa. (PDF) [file pntd.0011347.s001.pdf]

S1 Text

GEOFIL is a spatially explicit agent-based modelling framework, which was built to predict lymphatic filariasis (LF) transmission dynamics in American Samoa. GEOFIL builds and models a synthetic population in American Samoa that allows for births, deaths, couple formation, couple separation, moving within American Samoa, immigration, and emigration. Each human agent belongs to a family unit, which can be can be comprised of a couple or a single adult (divorced or unmarried), and possible children (including unmarried adult children). Households are generated from family units and as such, some households are composed of multiple family units. Aside from their household, people can be assigned, depending on their age, either a school or workplace. All households, workplaces, and schools have unique locations within the model that correspond to locations of known residential and non-residential buildings in American Samoa.

GEOFIL employs a radiation model [1], to predict daily commuting patterns. In the radiation model the flux from location i to j, T_{ij} is:

$$\langle T_{ij} \rangle = \frac{T_i m_i n_j}{(m_i + s_{ij})(m_i + n_j + s_{ij})} \quad (1)$$

where m_i and n_j are the populations of location i and j respectively, s_{ij} is the total population in radius r_{ij} around location i excluding n_i and n_j, and $T_i = \sum_{j \neq i} T_{ij}$ (the total flux of commuters from location i). The commuter model requires the distances between each location in the model, and for this distance, we used road distance instead of euclidean distance. This model allows workplaces to have workers from households all over the main island. However, as the single largest non-government employer in American Samoa is a tuna cannery located in the small village Atu'u (17.6% of the modelled work force are employed here), the radiation model was altered to account for the high flux of commuters into Atu'u. Unlike workplaces, students attend the school with the smallest euclidean distance from their household. All people aged 6-13 years attend elementary school, people aged 14-17 years attend secondary school (apart from a minority who are employed), and people aged 18-19 years who are not employed attend the American Samoa Community College. More details on the synthetic population model are provided in previous GEOFIL papers [2–4].

References

1. Simini F, González MC, Maritan A, Barabási AL. A universal model for mobility and migration patterns. Nature. 2012;484(7392):96–100. doi:10.1038/nature10856.

2. Xu Z, Glass K, Lau CL, Geard N, Graves P, Clements A. A synthetic population for modelling the dynamics of infectious disease transmission in American Samoa. Scientific reports. 2017;7(1):1–9. doi:10.1038/s41598-017-17093-8.

3. Xu Z, Lau CL, Zhou X, Fuimaono S, Magalhães RJS, Graves PM. The extensive networks of frequent population mobility in the Samoan Islands and their implications for infectious disease transmission. Scientific reports. 2018;8(1):1–11. doi:10.1038/s41598-018-28081-x.

4. Xu Z, Graves PM, Lau CL, Clements A, Geard N, Glass K. GEOFIL: A spatially-explicit agent-based modelling framework for predicting the long-term transmission dynamics of lymphatic filariasis in American Samoa. Epidemics. 2019;27:19–27. doi:10.1016/j.epidem.2018.12.003.
