# Supplementary material for: Lymphatic filariasis endgame strategies: Using GEOFIL to model mass drug administration and targeted surveillance and treatment strategies in American Samoa
Source: PLoS Negl Trop Dis. 2023 May 18;17(5):e0011347. doi: 10.1371/journal.pntd.0011347 (PMC10231811; doi:10.1371/journal.pntd.0011347)
Supplement: S2 Text — Description and underlying equations of the lymphatic filariasis transmission model. (PDF) [file pntd.0011347.s002.pdf]

S2 Text

In this study, for improved clarity, we have re-parameterised the transmission processes used in GEOFIL. The underlying dynamics of the processes are the same as in previous GEOFIL studies, however, we have re-named and combined various transmission parameters and processes. This re-parameterisation reduced the total number of parameters, and parameter names more accurately characterised what aspect of the transmission process each parameter represented.

The primary drivers of infections are the biting rate, the local prevalence in mosquitoes, and the probability that a bite from an infective mosquito will transmit third-stage (L3) larvae. Importantly, GEOFIL models the mosquito vector implicitly, while modelling the human-agent explicitly. The model is run on a daily time step, with worm status, transmission, and people's epidemiological status updated daily. In GEOFIL we calculate transmission during working hours and off-work hours separately. This is done as people have can be at different locations during working hours (workplace, school, or household) and off-work hours (household only), and because in American Samoa there are two primary vectors that bite at different times (*Aedes polynesiensis* during the day and *Aedes samoanus* at night). Therefore, total transmission is the sum of the transmission that occurs during these two periods.

During each time-step, a person has X transmission events, where X is a non-negative integer and a transmission event is an infective bite that deposit at least one L3 larvae that survives to maturity. The number of transmission events per time-step for each person is modelled as a Poisson random variable, with mean that depends on the local prevalence of infectious persons, as described in detail below. For simplicity, a transmission event passes either one L3 larvae of a single sex (probability p), or two L3 larvae with one each sex (probability $1 - p$). The transmitted larvae are *immature* and are assigned an immature period in days ($T_p \sim \mathcal{U}(182, 365)$) within their host person (the agent). If multiple worms are transmitted, all worms are assigned the same immature period. Worms at the end of their immature period are *mature*, and are assigned a mature period in days ($T_m \sim \mathcal{U}(4 \times 365, 6 \times 365)$). The mature worms die if they reach the end of their mature period, their host dies, or their host receives an effective treatment. While adult worms are modelled, this is only done to keep track of an individual's infection status due to potentially overlapping lifespans of multiple mature worms. To produce mf, and be infective, a person must have at least one mature worm of each sex. As the exact relationship between mature worm and mf burden is not clear, in GEOFIL we assume a persons' mf burden does not increase with additional pairs of mated worms. This is different to other LF modelling approaches e.g. EPIFIL, TRANSFIL, and LYMPHASIM, which assume a positive relationship between mating pairs of mature worms and mf burden.

The risk of infection is spatially heterogeneous as it is highly dependent on the local prevalence of infectious persons. L is the set of all locations (households, schools, and workplaces) in GEOFIL. For each location j, L_j ($L_j \subseteq L$) is the set of all locations within euclidean distance d_{max} of location j, where d_{max} is the maximum flight range of the vector. $M_{j,d}$ and $M_{j,n}$ are the sets of all persons at j during working hours and off-work hours respectively. The rate of transmission events for person i at location j during working hours is $c_i \mu_{j,d}$ and off-work hours is $c_i \mu_{j,n}$. The rates are proportional to the average mf prevalence in persons in L_j at time during working or off-work hours adjusted for the age-dependent relative biting exposure of person i (c_i), the distance

from location j, and the fertility of worms in host (which is affected by MDA):

$$\mu_{j,d/n} = \frac{\sum_{k \in L_j \cup \{j\}} \frac{f_d(j,k)}{N_k} \sum_{i \in M_{k,d/n}} c_i I_i}{\sum_{k \in L_j \cup \{j\}} f_d(j,k)} \times \beta_{d/n} \tag{1}$$

where $f_d(j,k) = 1 - \frac{d(j,k)}{d_{max}}$, $d(j,k)$ is the euclidean distance between locations j and k, I_i is the relative infectiousness of person i (accounting for sterilising effects of MDA and whether person i has a breeding pair of worms), and β_d or β_n are the transmission rates during work hours and off-work hours. The transmission rate parameters are not directly measurable and combine factors such as the biting rate, proportion of mosquito bites that are from mosquitoes that have previously had a blood meal, and efficacy of the vector. The magnitude of the total daily transmission rate (β_t), the ratio of the working hour transmission rate to the total daily transmission rate(β_d/β_t), and the proportion of transmission events that transfer one L3 larva (p) are all fitted parameters. Currently transmission rates differ between work and off-work hours but are not heterogeneous in space; however, if future studies provided spatial estimates of biting rates, $\beta_{d/n}$ could be updated to be temporally and spatially dependent.
