# Supplementary material for: Lymphatic filariasis endgame strategies: Using GEOFIL to model mass drug administration and targeted surveillance and treatment strategies in American Samoa
Source: PLoS Negl Trop Dis. 2023 May 18;17(5):e0011347. doi: 10.1371/journal.pntd.0011347 (PMC10231811; doi:10.1371/journal.pntd.0011347)
Supplement: S3 Text — Description of the model initialisation process. (PDF) [file pntd.0011347.s003.pdf]

S3 Text

GEOFIL is initialised with prevalence data from the 2010 American Samoa community survey [1] and due to limited survey data, we were unable to initialise with location specific prevalence. The survey found a territory-wide antigen prevalence of 3.2%. The survey also found higher antigen prevalence in persons aged ≥20 years than in persons aged <20 years. To initialise GEOFIL with this behaviour, the population is split into three age brackets and each age bracket was initialised with a different base antigen-prevalence. Persons aged ≥20 years had an antigen prevalence of 4.51%, while those aged between 5-19 had an antigen prevalence of 2.57% and those aged <5 years had 0% antigen prevalence. This gave a territory-wide base antigen prevalence of 3.2%.

For this study GEOFIL was updated to include mf case clustering at both the village and household level. As intra-cluster correlation (ICC) values were only calculated in a single survey (the 2016 community survey [2]), we assumed that household-level ICC and village-level ICC for mf-positivity was the same in 2010. Therefore, the model was initialised in 2010 with case-clustering to emulate both the household and village ICC values from the 2016 survey. As initialisation was random, and clustering introduced wide variability in the starting territory-wide prevalence around the target antigen prevalence of 3.2%, an initialisation was rejected if the territory-wide antigen prevalence fell outside of 2.75 -3.75%. This range was chosen to help reflect the uncertainty in the 2010 estimate of prevalence.

References

1. Lau CL, Won KY, Becker L, Magalhaes RJS, Fuimaono S, Melrose W, et al. Seroprevalence and spatial epidemiology of lymphatic filariasis in American Samoa after successful mass drug administration. PLoS Neglected Tropical Diseases. 2014;8(11):e3297. doi:10.1371/journal.pntd.0003297.

2. Lau CL, Sheel M, Gass K, Fuimaono S, David MC, Won KY, et al. Potential strategies for strengthening surveillance of lymphatic filariasis in American Samoa after mass drug administration: Reducing 'number needed to test'by targeting older age groups, hotspots, and household members of infected persons. PLoS Neglected Tropical Diseases. 2020;14(12):e0008916. doi:10.1371/journal.pntd.0008916.
