# Supplementary material for: Lymphatic filariasis endgame strategies: Using GEOFIL to model mass drug administration and targeted surveillance and treatment strategies in American Samoa
Source: PLoS Negl Trop Dis. 2023 May 18;17(5):e0011347. doi: 10.1371/journal.pntd.0011347 (PMC10231811; doi:10.1371/journal.pntd.0011347)
Supplement: S4 Text — Description of the approximate Bayesian computation fitting procedure used in the model. (PDF) [file pntd.0011347.s004.pdf]

S4 Text

We used approximate Bayesian computation (ABC) to fit GEOFIL to results of previous LF community surveys in American Samoa. The specific software we used for the ABC-fitting was ABCtoolbox [1]. The fitting itself used a simple fixed tolerance, with multiple runs for each parameter set to account for inter-run variability.

During the fitting the model was initialised in 2010, with the process described above, and run until 2016. As stated previously, in this study we re-parameterised the transmission side of GEOFIL for increased clarity. The ABC fitting was conducted on three parameters from the original parameterisation of GEOFIL, however, under simple transformations these parameters can be converted to the new parameterisation. We have included plots of both the original and the transformed priors and posteriors.

In the original parameterisation, the number of *bites*[1] a person could receive per day was constant (b_t), and these *bites* were split between *bites* during working hours (b_d) and *bites* during off-work hours (b_n). A *bite* did not guarantee transmission, rather there was a small probability a *bite* would transmit either one third-stage larva that would survive to maturity (p_1), or two third-stage larvae with one of each sex that would survive to maturity (p_2). It is important to note with this parameterisation, that from a *bite* a person received no larvae that survived to maturity with probability 1-p_1-p_2.

We fitted b_d, p_1 and p_2. We included the number of *bites* during working hours as it allowed for a shift in location where transmission primarily occurred, to ensure the model displayed the correct levels mf clustering at the village and household level. The probabilities that a bite would transmit either one or two third-stage larva, were included to fit the mf and antigen prevalences. Semi-informative uniform priors were used for all fitted parameters.

In the new parameterisation, the transformed fitted parameters were the magnitude of the total daily transmission rate (β_t), the ratio of the working hour transmission rate to the total daily transmission rate (β_d/β_t), and the proportion of transmission events that transfer one L3 larva (p). The transforms between the original and new parameters are:

$$\beta_t = \alpha(p_1 + p_2) \tag{1}$$

$$\frac{\beta_d}{\beta_t} = \frac{b_d}{b_t} \tag{2}$$

$$p = \frac{p_1}{p_1 + p_2} \tag{3}$$

where α is a constant that accounts for factors like proportion of mosquito bites that are from mosquitoes that have previously had a blood meal, biting rate, and efficacy of the vector.

We used both the 2014 and 2016 American Samoa LF community surveys [2, 3] to generate eight observed summary statistics. To ensure that simulated summary statistics were comparable to data, we emulated the 2014 and 2016 surveys in GEOFIL. In 2014 we used the total number of antigen positive people detected by the survey that were aged under 20, aged over 20, and the total number of people that were antigen positive who were also mf positive. For 2016, we used at the total number of antigen

[1]'Bites' is italicised because in GEOFIL as the mosquito vectors are only modelled implicitly, as such these biting rates should not be interpreted as an actual estimate on the mean number of bites received per person per day.

positive people detected by the survey that were aged under 18, aged over 18, the total number of people that were antigen positive who were also mf positive, the mf village level ICC, and the mf household level ICC.

The results of the ABC fitting are shown in S1 Fig. and S2 Fig. Due to computational limitations we were limited to 100 runs per strategy and as a consequence of this small number of replicates we decided to use point estimates (median values) and not samples from the posterior distribution for the three fitted parameters.

References

1. Wegmann D, Leuenberger C, Neuenschwander S, Excoffier L. ABCtoolbox: a versatile toolkit for approximate Bayesian computations. BMC bioinformatics. 2010;11(1):1–7.

2. Sheel M, Sheridan S, Gass K, Won K, Fuimaono S, Kirk M, et al. Identifying residual transmission of lymphatic filariasis after mass drug administration: Comparing school-based versus community-based surveillance-American Samoa, 2016. PLoS Neglected Tropical Diseases. 2018;12(7):e0006583. doi:10.1371/journal.pntd.0006583.

3. Lau CL, Sheel M, Gass K, Fuimaono S, David MC, Won KY, et al. Potential strategies for strengthening surveillance of lymphatic filariasis in American Samoa after mass drug administration: Reducing 'number needed to test'by targeting older age groups, hotspots, and household members of infected persons. PLoS Neglected Tropical Diseases. 2020;14(12):e0008916. doi:10.1371/journal.pntd.0008916.
