# Supplementary figures and images for: Lymphatic filariasis endgame strategies: Using GEOFIL to model mass drug administration and targeted surveillance and treatment strategies in American Samoa
Source: PLoS Negl Trop Dis. 2023 May 18;17(5):e0011347. doi: 10.1371/journal.pntd.0011347 (PMC10231811; doi:10.1371/journal.pntd.0011347)

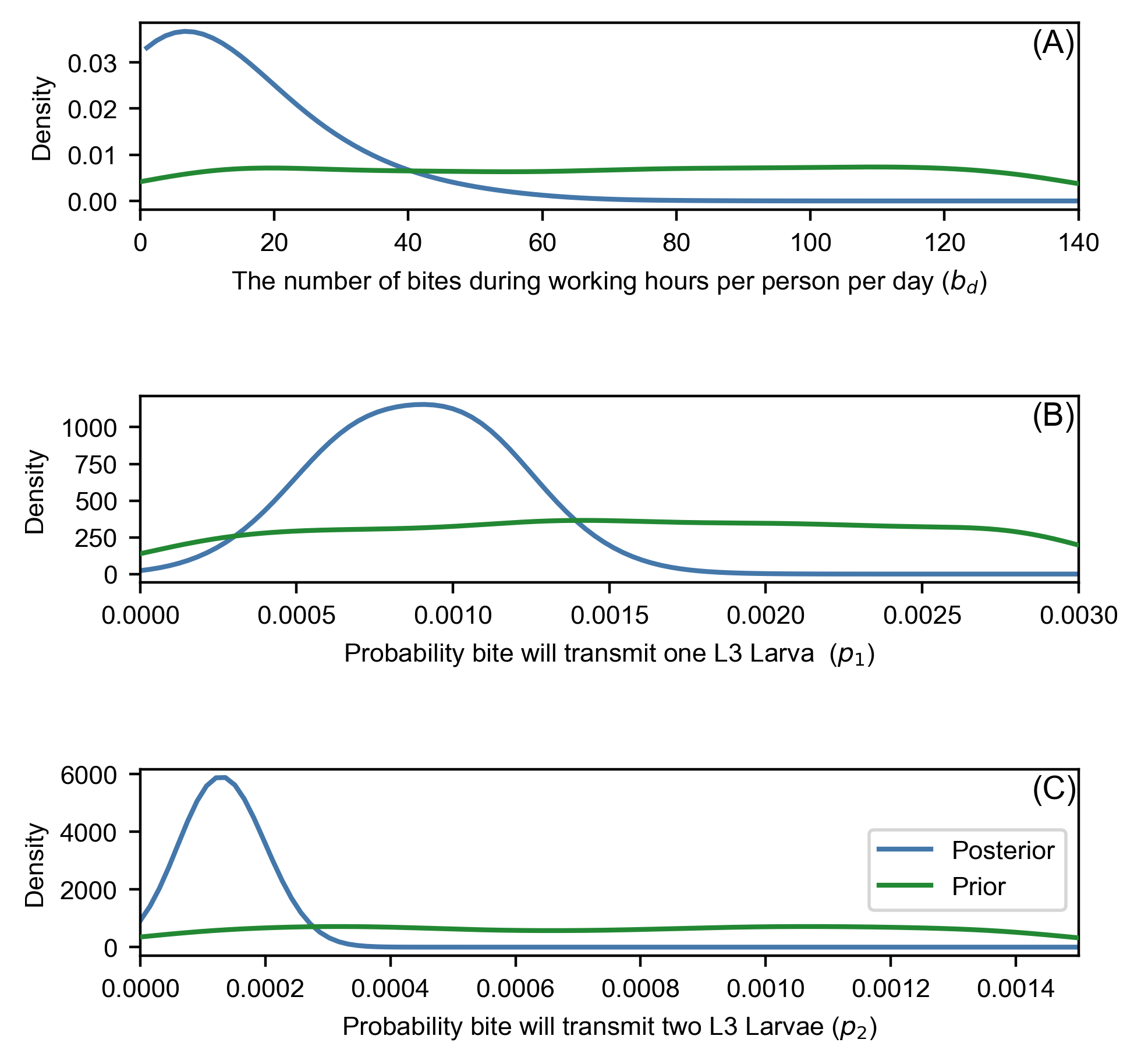

Supplement: S1 Fig — Priors and posterior distributions for the original three ABC-fitted parameters. Both the priors and posteriors are kernel-smoothed densities. (A) The mean number of bites received per person during working hours (bd). (B) The probability an infective bite would transmit one third-stage larva that will survive to maturity (p1). (C) The probability an infective bite would transmit two third-stage larvae with one of each sex that will survive to maturity (p2). (TIF) [file pntd.0011347.s005.tif]

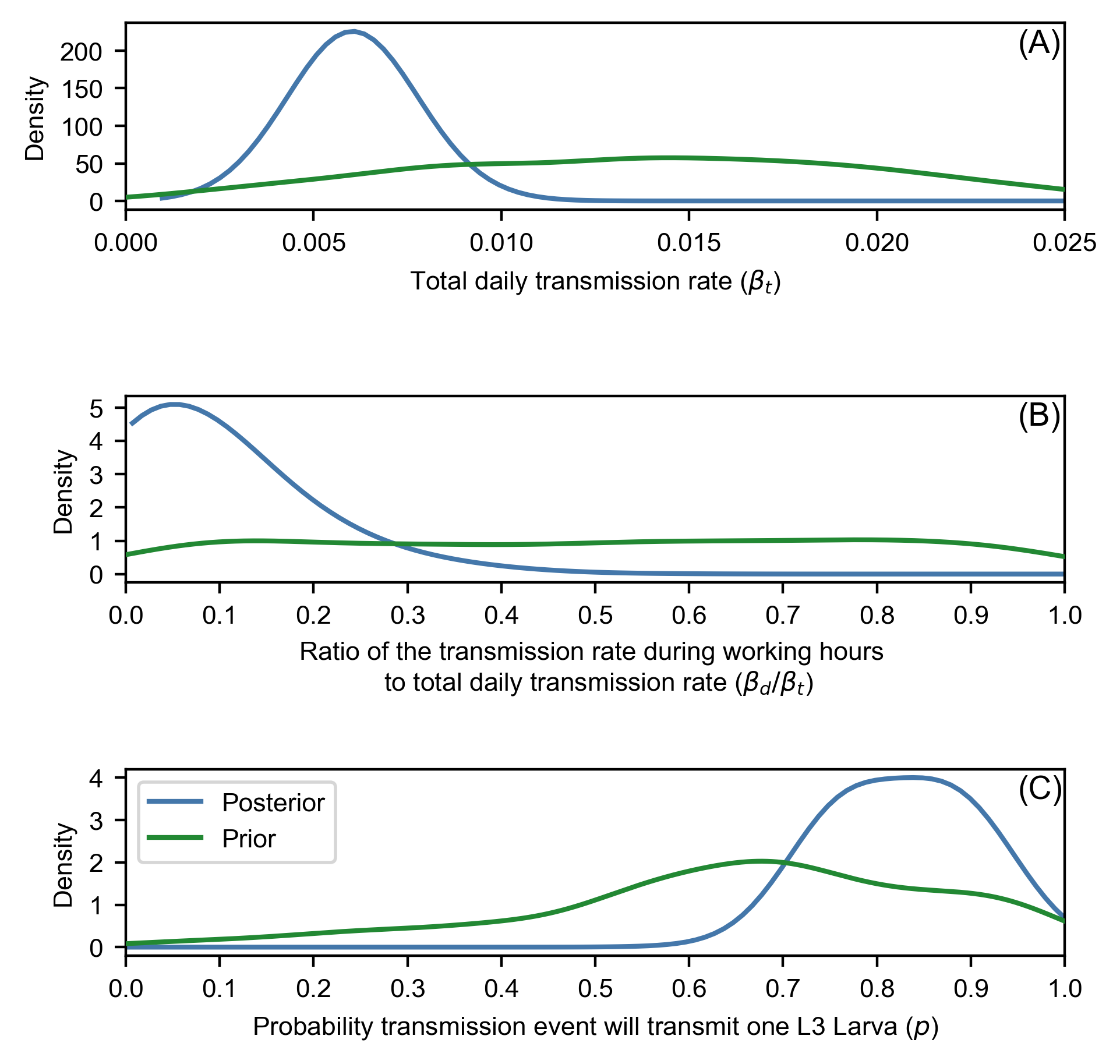

Supplement: S2 Fig — Prior and posterior distributions for the three transformed parameters. The priors and posteriors are kernel-smoothed densities. (A) The total daily transmission rate (βt). (B) The ratio of the working hour transmission rate to the total daily transmission rate (βd/βt). (C) The probability a transmission event will transmit one L3 larva (p). (TIF) [file pntd.0011347.s006.tif]

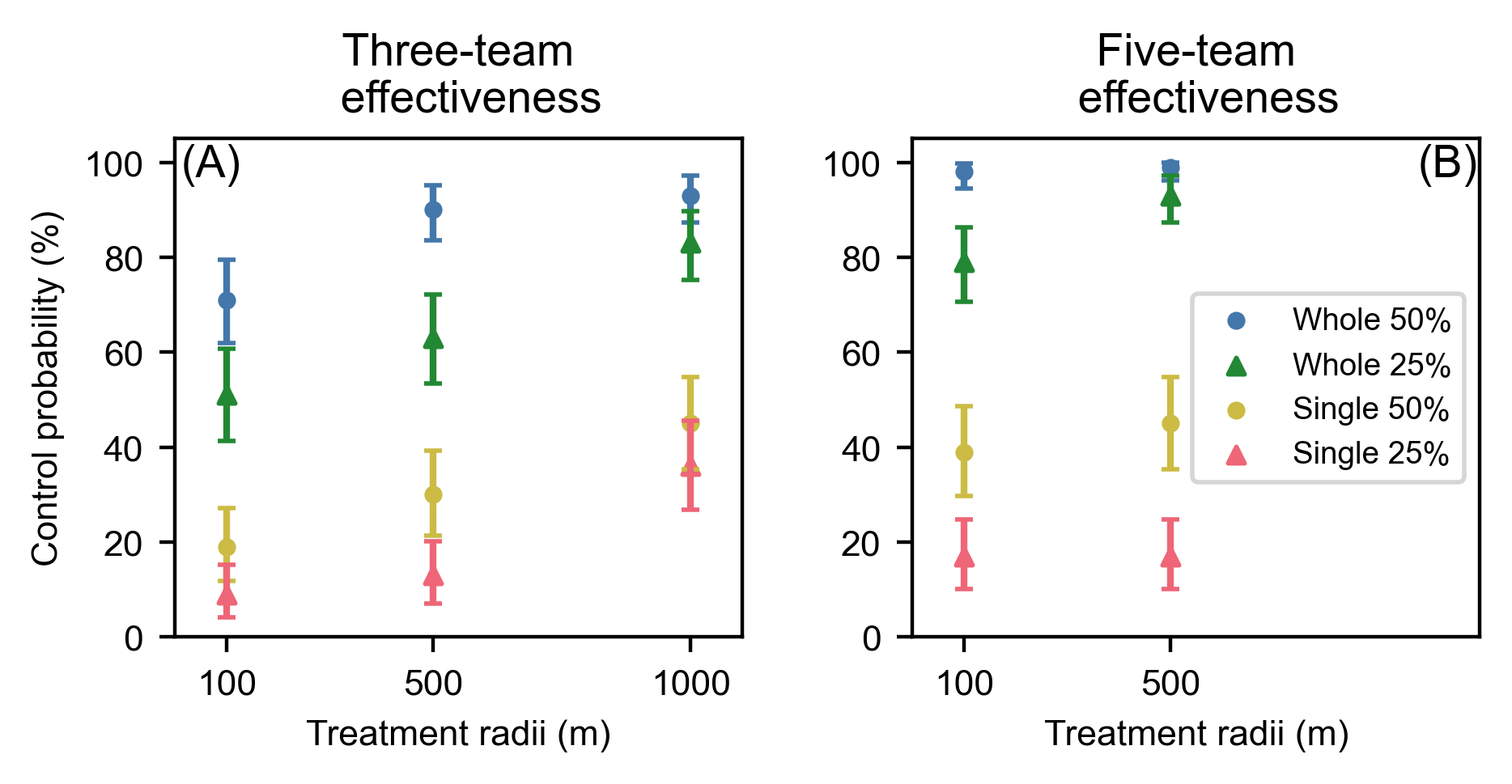

Supplement: S3 Fig — Comparison of the two household-based strategies where every household member was tested, and the modified household-based strategy where only a single member of a household was tested. As the testing of a single person was likely to find fewer mf or antigen positive persons than testing the entire household, we only modelled the single person testing with the most effective whole household testing strategies. (A) Comparison of the effectiveness of the three-team strategies. (B) Comparison of the effectiveness of the five-team strategies. (TIF) [file pntd.0011347.s007.tif]
