# Supplementary material for: Lymphatic filariasis endgame strategies: Using GEOFIL to model mass drug administration and targeted surveillance and treatment strategies in American Samoa
Source: PLoS Negl Trop Dis. 2023 May 18;17(5):e0011347. doi: 10.1371/journal.pntd.0011347 (PMC10231811; doi:10.1371/journal.pntd.0011347)
Supplement: S1 Table — Probability of territory-wide antigen prevalence falling below 1% at any time and control probability, for MDA, household-based strategies, and school- and workplace-based strategies. The uncertainties for the control probabilities and antigen <1% are quantified with a 95% credible interval. (PDF) [file pntd.0011347.s008.pdf]

S1 Table

Strategy	Probability >1% Antigen (%)	Control Probability (%)
School-TAS (6-7)	99 [96.2-100]	2 [0.2-5.5]
School-Elementary (6-13)	100 [98.1-100]	3 [0.5-7.0]
School-All (6-17)	100 [98.1-100]	4 [1.0-8.5]
Workplace 50+	100 [98.1-100]	0 [0-1.9]
Workplace 5+	100 [98.1-100]	3 [0.5-7.0]
Workplace 50+ & family	100 [98.1-100]	2 [0.2-5.5]
Workplace 5+ & family	100 [98.1-100]	13 [7.1-20.1]
MDA 65%- 1 Round	100 [98.1-100]	11 [5.6-17.6]
MDA 65%- 2 Round	100 [98.1-100]	48 [38.4-57.7]
MDA 65%- 3 Round	100 [98.1-100]	71 [61.9-79.5]
MDA 65%- 4 Round	100 [98.1-100]	94 [88.7-97.9]
MDA 65%- 5 Round	100 [98.1-100]	100 [98.1-100]
MDA 73%- 1 Round	100 [98.1-100]	23 [15.3-31.6]
MDA 73%- 2 Round	100 [98.1-100]	59 [49.3-68.4]
MDA 73%- 3 Round	100 [98.1-100]	93 [87.4-97.2]
MDA 73%- 4 Round	100 [98.1-100]	97 [93.0-99.5]
MDA 73%- 5 Round	100 [98.1-100]	100 [98.1-100]
MDA 85%- 1 Round	100 [98.1-100]	54 [44.3-63.6]
MDA 85%- 2 Round	100 [98.1-100]	90 [83.6-95.2]
MDA 85%- 3 Round	100 [98.1-100]	98 [94.5-99.8]
MDA 85%- 4 Round	100 [98.1-100]	100 [98.1-100]
MDA 85%- 5 Round	100 [98.1-100]	100 [98.1-100]
1 Team - 25% HTA - 100m	99 [96.2-100]	9 [4.1-15.2]
1 Team - 50% HTA - 100m	100 [98.1-100]	21 [13.6-29.3]
1 Team - 25% HTA - 500m	100 [98.1-100]	19 [11.9-27.1]
1 Team - 50% HTA - 500m	100 [98.1-100]	32 [23.2-41.3]
1 Team - 25% HTA - 1000m	100 [98.1-100]	28 [19.6-37.0]
1 Team - 50% HTA - 1000m	100 [98.1-100]	39 [29.7-48.6]
3 Team - 25% HTA - 100m	100 [98.1-100]	51 [41.3-60.7]
3 Team - 50% HTA - 100m	100 [98.1-100]	71 [61.9-79.5]
3 Team - 25% HTA - 500m	100 [98.1-100]	63 [53.5-72.1]
3 Team - 50% HTA - 500m	100 [98.1-100]	90 [83.6-95.2]
3 Team - 25% HTA - 1000m	100 [98.1-100]	83 [75.2-98.8]
3 Team - 50% HTA - 1000m	100 [98.1-100]	93 [87.4-97.2]
5 Team - 25% HTA - 100m	100 [98.1-100]	79 [70.7-86.4]
5 Team - 50% HTA - 100m	100 [98.1-100]	98 [94.5-99.8]
5 Team - 25% HTA - 500m	100 [98.1-100]	93 [87.4-97.2]
5 Team - 50% HTA - 500m	100 [98.1-100]	99 [96.2-100]
