# Supplementary material for: Lymphatic filariasis endgame strategies: Using GEOFIL to model mass drug administration and targeted surveillance and treatment strategies in American Samoa
Source: PLoS Negl Trop Dis. 2023 May 18;17(5):e0011347. doi: 10.1371/journal.pntd.0011347 (PMC10231811; doi:10.1371/journal.pntd.0011347)
Supplement: S2 Table — 3D-MDA/treatment (IDA) values are based upon the assumptions from Irvine et al [66]. (PDF) [file pntd.0011347.s009.pdf]

S2 Table

Variable	3D-MDA/ treatment (IDA)	2D-MDA/ treatment (DA)
Probability of worm death (%)	55	50
Probability of full worm sterilisation (%)	45	33
Probability of partial worm sterilisation (%)	0	17
Duration of sterilisation (years)	Permanent	1
