# Supplementary material for: Lymphatic filariasis endgame strategies: Using GEOFIL to model mass drug administration and targeted surveillance and treatment strategies in American Samoa
Source: PLoS Negl Trop Dis. 2023 May 18;17(5):e0011347. doi: 10.1371/journal.pntd.0011347 (PMC10231811; doi:10.1371/journal.pntd.0011347)
Supplement: S3 Table — (PDF) [file pntd.0011347.s010.pdf]

S3 Table

Parameter	Value	Description	Reference
d_{max}	100m	Maximum flight range of *Aedes* mosquitos	[1,2]
l_m	13 days	Length of extrinsic incubation period	[3]
l_p	6-12 months	Length of immature period	[4]
l_l	4-6 years	Length of mature period	[4]
s_m	0.6	Mosquito survival probability of each feeding cycle (3 days)	[5]
p_{L3}	$s_m^{l_m/3}$	Probability of mosquitoes surviving through the extrinsic incubation period	-
p_{if}	0.3881	Probability that mosquitoes which survive through the extrinsic incubation period are infective	[6,7]
b_t	140	Total daily *bites*	[1,8,9]
α	$b_t \times p_{L3} \times p_{if}$	Factor to account for biting rate and the efficacy of the vector	-
c	0-1	Relative mosquito exposure of individuals aged ≤ 15 years. Kernel smoothed based on: 0.25 (0–4 year-olds), 0.75 (5–15 year-olds)	[10]
p	0.869	Probability that a transmission event transmits a single worm	Fitted
$1-p$	0.131	Probability that a transmission event transmits both a male and female worm	Fitted
β_t	6.03×10^{-3}	Total daily transmission rate	Fitted
β_d	6.27×10^{-4}	Working hours transmission rate	Fitted
β_n	β_t - β_d	Off-work hours transmission rate	Fitted

References

1. Hapairai LK, Sang MAC, Sinkins SP, Bossin HC. Population studies of the filarial vector Aedes polynesiensis (Diptera: Culicidae) in two island settings of French Polynesia. Journal of medical entomology. 2013;50(5):965–976. doi:10.1603/ME12246.

2. Lau CL, Won KY, Lammie PJ, Graves PM. Lymphatic filariasis elimination in American Samoa: evaluation of molecular xenomonitoring as a surveillance tool in the endgame. PLoS Neglected Tropical Diseases. 2016;10(11):e0005108. doi:10.1371/journal.pntd.0005108.

3. Paily K, Hoti S, Das P. A review of the complexity of biology of lymphatic filarial parasites. Journal of Parasitic Diseases. 2009;33(1):3–12. doi:10.1007/s12639-009-0005-4.

4. Ottesen EA. Lymphatic filariasis: treatment, control and elimination. Advances in parasitology. 2006;61:395–441. doi:10.1016/S0065-308X(05)61010-X.

5. Graves P, Burkot T, Saul A, Hayes R, Carter R. Estimation of anopheline survival rate, vectorial capacity and mosquito infection probability from malaria vector infection rates in villages near Madang, Papua New Guinea. Journal of Applied Ecology. 1990; p. 134–147. doi:10.2307/2403573.

6. Krishnamoorthy K, Subramanian S, Van Oortmarssen G, Habbema J, Das P. Vector survival and parasite infection: the effect of Wuchereria bancrofti on its vector Culex quinquefasciatus. Parasitology. 2004;129(1):43–50. doi:10.1017/S0031182004005153.

7. Erickson SM, Thomsen EK, Keven JB, Vincent N, Koimbu G, Siba PM, et al. Mosquito-parasite interactions can shape filariasis transmission dynamics and impact elimination programs. PLoS neglected tropical diseases. 2013;7(9):e2433. doi:10.1371/journal.pntd.0002433.

8. Jachowsji Jr LA. Filariasis in American Samoa: V. Bionomics of the principal vector, Aedes polynesiensis Marks. American Journal of Epidemiology. 1954;60(2):186–203. doi:10.1093/oxfordjournals.aje.a119712.

9. Ramalingam S. The epidemiology of filarial transmission in Samoa and Tonga. Annals of Tropical Medicine & Parasitology. 1968;62(3):305–324. doi:10.1080/00034983.1968.11686565.

10. Stone W, Gonçalves BP, Bousema T, Drakeley C. Assessing the infectious reservoir of falciparum malaria: past and future. Trends in parasitology. 2015;31(7):287–296. doi:10.1016/j.pt.2015.04.004.
