# Supplementary material for: Lymphatic filariasis endgame strategies: Using GEOFIL to model mass drug administration and targeted surveillance and treatment strategies in American Samoa
Source: PLoS Negl Trop Dis. 2023 May 18;17(5):e0011347. doi: 10.1371/journal.pntd.0011347 (PMC10231811; doi:10.1371/journal.pntd.0011347)
Supplement: Attachment — Submitted filename: Review_Response_Letter-1.pdf [file pntd.0011347.s011.pdf]

Dear Prof. Basáñez and Prof. Cantacessi,

Thank you for considering our manuscript for publication in *PLOS Neglected Tropical Diseases* and organising the feedback of the two reviewers. We would particularly like to thank the reviewers for their time and effort in providing feedback on the manuscript. Please find below a detailed list of responses for the comments of each reviewer, with actions in **bold**.

Reviewer 1

1. *The 'Methods' section needs a major revision, as the model, fitting method, and prevalence data are not clearly described...*

   We agree with the reviewer that not enough information on the model, ABC-fitting, and prevalence data was included in the original manuscript. To rectify this, we have included more information on these areas in both the methods section and in the appendix. **Additional information on the GEOFIL model, fitting method, and data used in the fitting has been included in the methods and appendix sections of the revised manuscript.**

2. *The authors have included 'probability of control and the number of tests and treatments' (line 257) and state that the results are summarized in Figure 4. However, I couldn't find Figure 4 in the manuscript.*

   Figure 4 was mistakenly not included in the manuscript. **Figure 4 now included in the manuscript.**

3. *In the Abstract and throughout the manuscript, I would suggest using IDA for the triple drug as opposed to 3D-MDA. Similar comment for 2D-MDA which can be IA or DA. By using 2D-MDA it is not clear which treatment was used (IA or DA?).*

   We thank the reviewer for raising this point. We have decided to keep the original notation of 3D-MDA and 2D-MDA, when referring to MDA treatment, however, we have clarified their usage in the manuscript to remove the ambiguity around 2D-MDA. To avoid confusion when discussing the targeted strategies, treatments are now referred to as '3D-treatment' for IDA and '2D-treatment' for DA. **The use of 2D-MDA and 3D-MDA has been clarified to avoid ambiguity around IA and DA. While the notation for the treatment used in the targeted strategies has been changed.**

4. *In the Abstract, clarify whether the coverage used is for the whole population or for the eligible population.*

   The coverage was for the total population, but only the eligible population could receive treatment. **The metric used for coverage has been clarified in the manuscript.**

5. *In the Abstract, regarding the statement: 'All interventions were finished. . . ', the author should clarify that these are simulated interventions?*

   These were simulated interventions. **This has been clarified in the manuscript.**

6. *Line 49: Is this elimination of transmission (EoT) or elimination as a public health problem (EPHP)?*

   Elimination in this context was elimination as a public health problem. **The use of 'elimination' in the section of the manuscript has been clarified.**

7. *Line 78: 'However, alternative strategies should...', this is a statement that needs additional explanation. Which are these alternative strategies and what is their impact on the elimination target?*

   We agree with the reviewer that this statement was vague. Our intention was to highlight the potential use of control strategies other than territory-wide MDA in a low prevalence setting. These targeted interventions, raised later in the introduction, rely on targeted surveillance. Previous studies, including one in American Samoa [1], have found surveillance of certain sub-populations is more effective (requires fewer tests to find a positive case) in identifying residual infections in a low prevalence setting. Targeted surveillance could be used to inform targeted interventions, which could be less burdensome than territory-wide MDA. Importantly, we are not making a statement about the effect of these targeted interventions on the threshold behaviour, rather that these strategies may allow the threshold prevalence to be reached more efficiently. **We have changed this statement in the manuscript to give it additional context.**

8. *Line 97: Briefly describe the model.*

   We have included a short description of the GEOFIL model in the methods section.

9. *Line 100: Give more information on the data used (i.e, demography, prevalence, etc). Were these data collected 4 years after the last MDA? and Line 103-108: Give more details on the fitting method.*

   GEOFIL is initialised with prevalence data from the 2010 American Samoa community survey [2]. This survey was conducted four years after the previous round of MDA. The survey found a territory-wide antigen prevalence of 3.2%. The survey also found higher antigen prevalence in persons aged $\geq$20 years than in persons aged $<$20 years. To initialise GEOFIL with this behaviour, the population is split into three age brackets and each age bracket was initialised with a different base antigen-prevalence. For this manuscript, GEOFIL was updated to include mf case clustering at both the village and household level. As household and village level intra-cluster correlation (ICC) could only be calculated in a single survey (the 2016 community survey [1]), we assumed that household-level ICC and village-level ICC for mf-positivity was the same in 2010.

   We used approximate Bayesian computation (ABC) to fit GEOFIL to results of previous LF community surveys in American Samoa. The specific software we used for the ABC-fitting was ABCtoolbox [3]. The fitting itself used a simple fixed tolerance, with multiple runs for each parameter set to account for inter-run variability. For the fitting, the model was initialised in 2010, with the process described above, and run until 2016.

   We included three parameters in the ABC fitting, defined as follows. The number of *bites* (bites is italicised as the mosquito-vector is only modelled implicitly) a person could receive per day was constant (b_t), and these *bites* were split between *bites* during working hours (b_d) and *bites* during off-work hours (b_n). A *bite* did not guarantee transmission, rather there was a small probability a *bite* would transmit either one third-stage larva that would survive to maturity (p_1), or two third-stage larvae with one of each sex that would survive to maturity (p_2). Parameters b_d, p_1 and p_2 were the three parameters included in the ABC fitting. We used b_d as it allowed for a shift in location where transmission primarily occurred, to ensure the model displayed the correct levels mf clustering at the village and household level. p_1 and p_2 were included to fit the mf and antigen prevalences. Semi-informative uniform priors were used for all fitted parameters.

   We used both the 2014 and 2016 American Samoa LF community surveys [1, 4] to generate eight observed summary statistics. To ensure that simulated summary statistics were comparable to data, we emulated the 2014 and 2016 surveys in GEOFIL.

Due to computational limitations we were limited to 100 runs per strategy and as a consequence of this small number of replicates we decided to use point estimates and not samples from the posterior distribution for the three fitted parameters. **We included a more detailed explanation of initialisation and the fitting process in the supplementary materials.**

10. *Line 185: Why 100 runs?*

    Due to computational restraints we were limited to 100 runs per strategy. Ideally we would have run each strategy more than 100 times, however, 100 runs took approximately eight hours to complete and there were 39 different strategies modelled. Due to the low number of replicates the results should be presented with the necessary estimates of uncertainty. **The probability of control results have been updated to include uncertainty.**

11. *Regarding Figure S1: Fix the x-axis, 1e-3 looks odd at the end. Add more values on the y-axis (for the density).*

    The 1e-3 has been moved and more values on the y-axis have been included.

12. *Line 216: Regarding Figure 3: Add a horizontal line at 1% threshold. Also, add more values on the x-axis. Currently, the x-axis has the years 2020 and 2030 only.*

    Figure has been updated to add more years on the x-axis and to also a horizontal line at the 1% thresholds.

13. *General comment about the figures: Please follow the journal's guideline when formatting them.*

    We have endeavoured to follow all guidelines, and are happy to make any specific changes required to adhere to the journal's guidelines.

Reviewer 2

For clarity we have broken up the second reviewer's primary comment point by point.

1. *The transmission model is not described, and is also poorly described in the reference given for the model's origin*

   We thank the reviewer for raising this point. While the transmission model was not described in this paper, it is described in detail in the original GEOFIL papers, particularly the appendix of latest published article paper. **For transparency and clarity we have expanded on sections of the methods and have included a detailed description of the transmission model as an appendix to this manuscript.**

   - *Mechanism for sexual reproduction...*

     While the original version of GEOFIL [5] only modelled the life cycle of mated pairs of worms, in the most recent publication GEOFIL has been extended to include individual male and female worms, as described in detail in the appendix to that manuscript [6]. We have retained the same transmission framework in the current work. In the model, a transmission event can either transmit L3 larvae of a single sex, or L3 larvae of both sexes. Transmitted worms are then assigned an immature period and if multiple worms, all worms are assigned the same immature period.

At the end of their immature period worms are then assigned a mature period. The mature worms can die if they reach the end of their mature period, their human host dies, or their host receives an effective dose of either ivermectin, diethylcarbamazine, and albendazole or diethylcarbamazine and albendazol. To produce mf, and be infective, a person must have at least one worm of each sex in their mature phase. Drugs can also sterilise adult worms preventing or reducing transmission.

To account for the sex and maturity of worms during initialisation, if a person was initialised with an LF infection there is a 78.5% probability they have one mature worm of one gender (not infectious), 14.3% probability of one mature worm of each gender (infectious), and 7.2% probability they are initialised with two immature worms, one of each gender (not infectious).

- *a form of negative density dependence...*

  As the exact relationship between adult worm and mf burden is not clear, in GEOFIL we make the parsimonious assumption that people's mf burden does not increase with additional pairs of mated worms. This introduces the required negative density dependence at the individual level.

- *Heterogeneity in exposure to biting...*

  We agree with the reviewer that this heterogeneity in exposure to biting is an important factor to consider. In the absence of appropriate data to inform geographic differences in biting rate, GEOFIL we assume a homogeneous biting rate across American Samoa. However, the high-level geographic information included in the model allows for heterogeneous distribution in transmission events.

  In GEOFIL we calculate transmission during working hours and off-work hours separately. This is done as people have can be at different locations during working hours (workplace, school, or household) and off-work hours (household only), and because in American Samoa there are two primary vectors that bite at different times (*Aedes polynesiensis* during the day and *Aedes samoanus* at night). The number of transmission events at each time-step for each person are modelled as Poisson random variables, with mean that depends on the time-dependent local prevalence of infectious persons.

2. *The paper also needs more detail on how the model is fitted to data in particular the precise form of the summary statistic used in the ABC procedure, and which form of ABC is being used...*

   Please see response to comment 9 from reviewer 1.

3. *It also isn't clear whether simulations are run using samples from the posterior distribution of parameters or from point estimates, or if the model is assumed to be stationary at the beginning of the simulations in 2010.*

   Due to computational limitations we were limited to 100 runs per strategy and as a consequence of this small number of replicates we decided to use point estimates and not samples from the posterior distribution for the three fitted parameters. We are also unsure what the reviewer meant by 'stationary' in 2010. As American Samoa is in a post-MDA scenario the model is not at endemic equilibrium in 2010. If the reviewer is asking about the initialisation process, the model population and distribution of infections in this population were re-initialised every run, therefore no two runs had identical populations or LF prevalences. **We have updated the section on model fitting to state that we used point estimates of parameters for simulations of interventions.**

4. *I also have concerns about the low number of replicates used to generate results, which appears to*

be 100. Given this low number, the results should be presented with an estimate of uncertainty

Please see response to comment 10 from reviewer 1.

5. *Given the spatial aspect of the study, it might be a nice addition to see maps of prevalence (if possible).*

   While it would be possible to generate prevalence maps, we have decided to not include any as this paper focuses on American Samoa as a whole, not predictions for specific locations within American Samoa. Also, due to the limitation of survey data available we were unable to initialise and fit the model with location specific prevalence, instead initialising and fitting to the macroscopic properties of disease distribution (i.e. prevalence of Ag and mf by age group, and ICC at household and village levels). Any predictions of prevalence in specific locations would be quite dependent on initial conditions and have a high degree of random variability over the course of simulation. While prevalence maps from an individual run would be useful for illustrative purposes, it risks misleadingly suggesting that the model is suited for predicting prevalence in specific locations.

References

[1] Colleen L Lau et al. "Potential strategies for strengthening surveillance of lymphatic filariasis in American Samoa after mass drug administration: Reducing 'number needed to test'by targeting older age groups, hotspots, and household members of infected persons". In: *PLoS Neglected Tropical Diseases* 14.12 (2020), e0008916. DOI: 10.1371/journal.pntd.0008916.

[2] Colleen L Lau et al. "Seroprevalence and spatial epidemiology of lymphatic filariasis in American Samoa after successful mass drug administration". In: *PLoS Neglected Tropical Diseases* 8.11 (2014), e3297. DOI: 10.1371/journal.pntd.0003297.

[3] Daniel Wegmann et al. "ABCtoolbox: a versatile toolkit for approximate Bayesian computations". In: *BMC bioinformatics* 11.1 (2010), pp. 1–7.

[4] Meru Sheel et al. "Identifying residual transmission of lymphatic filariasis after mass drug administration: Comparing school-based versus community-based surveillance-American Samoa, 2016". In: *PLoS Neglected Tropical Diseases* 12.7 (2018), e0006583. DOI: 10.1371/journal.pntd.0006583.

[5] Zhijing Xu et al. "GEOFIL: A spatially-explicit agent-based modelling framework for predicting the long-term transmission dynamics of lymphatic filariasis in American Samoa". In: *Epidemics* 27 (2019), pp. 19–27. DOI: 10.1016/j.epidem.2018.12.003.

[6] Angus McLure et al. "Modelling lymphatic filariasis elimination in American Samoa: GEOFIL predicts need for new targets and six rounds of mass drug administration". In: *Epidemics* (2022), p. 100591. ISSN: 1755-4365. DOI: 10.1016/j.epidem.2022.100591.
