# Supplementary material for: Lymphatic filariasis endgame strategies: Using GEOFIL to model mass drug administration and targeted surveillance and treatment strategies in American Samoa
Source: PLoS Negl Trop Dis. 2023 May 18;17(5):e0011347. doi: 10.1371/journal.pntd.0011347 (PMC10231811; doi:10.1371/journal.pntd.0011347)
Supplement: Attachment — Submitted filename: Review_Response_Letter_R2-1.pdf [file pntd.0011347.s013.pdf]

Dear Prof. Basáñez and Prof. Cantacessi,

Thank you for considering our manuscript for publication in *PLOS Neglected Tropical Diseases* and organising the feedback from the two reviewers. We would particularly like to thank both reviewers for their time and the second reviewer for providing another round of feedback on the manuscript. Note that the first reviewer did not suggest any further changes. Please find below a detailed list of responses for the second reviewer, with actions in **bold**.

Reviewer 2

1. *Is there a reason that worms are initialised in this way, is it simply to approximate the prevalence of mf positive people in the data set, or is based on some other data?*

   The model was initialised this way in order to approximate the mf and antigen positive prevalence observed in the previous community surveys in American Samoa.

2. *I agree that the exact relationship is not clear, and this approach does create the negative density dependence required, although a person having 100 mating pairs having the same mf concentration in blood as a person with 1 seems unrealistic to me. The standard approach in the literature has a linear relationship between the two, and the source of negative density dependence is in the mosquitos uptake of mf. However, it is a good thing that there is a diversity of modelling approaches so I'm fine with this. Nevertheless, I would recommend that the text here mentions that this approach differs to others' approaches e.g. EPIFIL, TRANSFIL, LYMPHASIM etc...*

   We included a line in the appendix highlighting that the this relationship between mf burden and mating pairs is different to the relationship traditionally used by other modelling approaches.

3. *Heterogeneity is not only a spatial feature to consider, but also a local one. Measured mf count distributions at local scales are also typically overdispersed/aggregated (higher variance-to-mean than a Poisson distribution) and this has an effect on the transmission dynamics. It appears the model has no mechanism to produce this phenomenon?*

   In GEOFIL, while we do model adults worms, this is only done to keep track of infection status of an individual due to potentially overlapping lifespans of multiple adult worms. We do not model an individual's mf burden explicitly, as we assume that individuals have equally infectiousness no matter the number of mated pairs.

   We agree that heterogeneity in probability of carriage is important. Spatial heterogeneity and local transmission are linked closely in GEOFIL. The daily risk of infection (transmission events) is determined at the building scale. If a majority of an individual's neighbours are infectious, they will likely have a higher rate of exposure and burden of adult worms than an individual of the same age in a neighbourhood where there are few or no infections. In other words, GEOFIL induces clustering in burden as exposure to transmission events is heterogeneous because prevalence in humans within transmission distance is heterogeneous. While household-level risk is already a very fine scale of resolution, there are also differences within households: e.g. daily risk of infection is lower for children because they are bitten less often, and overall prevalence of infection is higher in adults because of their cumulative exposure over the years. All of these factors would tend to a produced over-dispersed counts of adult worms in hosts. **We further clarified how mf and transmission events are handled in GEOFIL**

4. *This explanation of the heterogeneous distribution of transmission events makes it sound like the*

only source of spatial heterogeneity comes from the locations of work/school/household – are other geographic factors such as mosquito habitat making some locations inherently more risky than others, something that should be considered?

The reviewer is correct; currently the only source of transmission heterogeneity in the model is from building locations and we do not include other geographic factors. We had considered taking the suggested approach; however, the small geographic area of American Samoa and potential data set resolution pose a challenge, as a previous study into environmental factors associated with Lymphatic Filariasis infection in American Samoa was affected by data resolution [1]. However, including geographic features, provided they are of high enough resolution and can be appropriately parameterised in the model, would be worthwhile future extension to the model to improve predictions. Note however that we are not attempting to make spatially explicit predictions in our models (e.g. prevalence in specific villages or households). The rationale to include spatial structure is to understand how this effects transmission dynamics and enable modelling of spatially-informed interventions such as the snowball modelling.

5. *It appears that the implicit reason given here for limiting to 100 runs, though not explicitly stated, is that the authors believe that this will introduce more uncertainty into the results, because there is no extra computational cost to sampling from the posterior. My personal view is I don't think this is a good reason to throw away the uncertainty – this can give the impression of false precision. I am willing to accept this, though, as now the manuscript is transparent that they are not using the posteriors. I do note, however, that the manuscript does not say which point estimates are used – is it the MAP, mean or median?*

   We agree with the reviewer that running simulations with parameter values sampled from the posterior would be a good idea. We will certainly consider the option for future work. It would be possible, as the reviewer suggests, to sample from the posterior predictive distribution without increasing computation time by running one simulation each for 100 draws from the posterior distribution. One concern would be that this would not be sufficient to explore both the uncertainty in parameters and the uncertainty introduced by the stochastic nature of model initialisation, infection dynamics, and interventions and that it would be necessary to run multiple simulations per draw from the posterior and/or increase the numbers of draws from the posterior. Given computational limitations, we believe it is better (but not ideal) to explore a smaller subset of the uncertainty more fully and be transparent that we are not exploring all sources of uncertainty. We are thankful that the reviewer thinks that this course is also acceptable.

   With regards to point estimates of parameters, we used and reported posterior median values. **This has been clarified in the manuscript.**

6. *If it is not possible due to the nature of the data that is unfortunate but understandable. However, if spatial models are to have utility over simpler models, then it will be in their ability to give spatial predictions, as it is conceivable that a simpler model will be able to fit the aggregated data equally as well, and also give accurate predictions. I think in the discussion it should be stated that making spatial predictions that are accurate is at least an ambition.*

   We agree with the reviewer that there is utility in the ability to make spatial predictions for transmission (e.g. predicted risk surfaces). In the future if there were higher resolution location specific surveys, we may have the ability to make these kind of predictions with later evolutions of GEOFIL. However, data limitations and computational complexity of fitting models to spatially explicit prevalence data would be major obstacles. Nevertheless we believe there is still utility to spatial models even if they don't result in something like a risk surface. Namely, we believe the exposure heterogeneity introduced by spatial structure of the model is different to other model's approaches to model exposure heterogeneity with possible implications for notions like 'critical

prevalence thresholds'. Furthermore, our model structure allows simulation of interventions like snowball sampling or the household-based intervention modelled in this manuscript, that could not be simulated by other models.

We included a line in the manuscript highlighting that the inclusion of more geographic factors is an eventual goal of later evolutions of GEOFIL

References

[1] Morgan E Lemin et al. "Spatially Explicit Environmental Factors Associated with Lymphatic Filariasis Infection in American Samoa". In: *Tropical Medicine and Infectious Disease* 7.10 (2022), p. 295.
