# Supplementary material for: Lymphatic filariasis endgame strategies: Using GEOFIL to model mass drug administration and targeted surveillance and treatment strategies in American Samoa
Source: PLoS Negl Trop Dis. 2023 May 18;17(5):e0011347. doi: 10.1371/journal.pntd.0011347 (PMC10231811; doi:10.1371/journal.pntd.0011347)
Supplement: Attachment — Submitted filename: reviewLFpaper.docx [file pntd.0011347.s012.docx]

Reviewer 2

For clarity we have broken up the second reviewer’s primary comment point by point.

1. *The transmission model is not described, and is also poorly described in the reference given for the model’s origin*

We thank the reviewer for raising this point. While the transmission model was not described in this paper, it is described in detail in the original GEOFIL papers, particularly the appendix of latest published article paper. For transparency and clarity we have expanded on sections of the methods and have included a detailed description of the transmission model as an appendix to this manuscript.

This is a large improvement, the model is now much more clearly specified.

*• Mechanism for sexual reproduction...*

While the original version of GEOFIL [5] only modelled the life cycle of mated pairs of worms, in the most recent publication GEOFIL has been extended to include individual male and female worms, as described in detail in the appendix to that manuscript [6]. We have retained the same transmission framework in the current work. In the model, a transmission event can either transmit L3 larvae of a single sex, or L3 larvae of both sexes. Transmitted worms are then assigned an immature period and if multiple worms, all worms are assigned the same immature period. 3 At the end of their immature period worms are then assigned a mature period. The mature worms can die if they reach the end of their mature period, their human host dies, or their host receives an effective dose of either ivermectin, diethylcarbamazine, and albendazole or diethylcarbamazine and albendazol. To produce mf, and be infective, a person must have at least one worm of each sex in their mature phase. Drugs can also sterilise adult worms preventing or reducing transmission. To account for the sex and maturity of worms during initialisation, if a person was initialised with an LF infection there is a 78.5% probability they have one mature worm of one gender (not infectious), 14.3% probability of one mature worm of each gender (infectious), and 7.2% probability they are initialised with two immature worms, one of each gender (not infectious).

Is there a reason that worms are initialised in this way, is it simply to approximate the prevalence of mf positive people in the data set, or is based on some other data?

*• a form of negative density dependence...*

As the exact relationship between adult worm and mf burden is not clear, in GEOFIL we make the parsimonious assumption that people’s mf burden does not increase with additional pairs of mated worms. This introduces the required negative density dependence at the individual level.

I agree that the exact relationship is not clear, and this approach does create the negative density dependence required, although a person having 100 mating pairs having the same mf concentration in blood as a person with 1 seems unrealistic to me. The standard approach in the literature has a linear relationship between the two, and the source of negative density dependence is in the mosquitos uptake of mf. However, it is a good thing that there is a diversity of modelling approaches so I’m fine with this. Nevertheless, I would recommend that the text here mentions that this approach differs to others’ approaches e.g. EPIFIL, TRANSFIL, LYMPHASIM etc…

*• Heterogeneity in exposure to biting...*

We agree with the reviewer that this heterogeneity in exposure to biting is an important factor to consider. In the absence of appropriate data to inform geographic differences in biting rate, GEOFIL we assume a homogeneous biting rate across American Samoa. However, the high-level geographic information included in the model allows for heterogeneous distribution in transmission events. In GEOFIL we calculate transmission during working hours and off-work hours separately. This is done as people have can be at different locations during working hours (workplace, school, or household) and off-work hours (household only), and because in American Samoa there are two primary vectors that bite at different times (Aedes polynesiensis during the day and Aedes samoanus at night). The number of transmission events at each time-step for each person are modelled as Poisson random variables, with mean that depends on the time-dependent local prevalence of infectious persons.

There are two points to address here. 1. Heterogeneity is not only a spatial feature to consider, but also a local one. Measured mf count distributions at local scales are also typically overdispersed/aggregated (higher variance-to-mean than a Poisson distribution) and this has an effect on the transmission dynamics. It appears the model has no mechanism to produce this phenomenon? 2. This explanation of the heterogeneous distribution of transmission events makes it sound like the only source of spatial heterogeneity comes from the locations of work/school/household – are other geographic factors such as mosquito habitat making some locations inherently more risky than others, something that should be considered?

2. *The paper also needs more detail on how the model is fitted to data in particular the precise form of the summary statistic used in the ABC procedure, and which form of ABC is being used...*

Please see response to comment 9 from reviewer 1.

There fitting procedure is now well explained.

3. *It also isn’t clear whether simulations are run using samples from the posterior distribution of parameters or from point estimates, or if the model is assumed to be stationary at the beginning of the simulations in 2010.*

Due to computational limitations we were limited to 100 runs per strategy and as a consequence of this small number of replicates we decided to use point estimates and not samples from the posterior distribution for the three fitted parameters. We are also unsure what the reviewer meant by ‘stationary’ in 2010. As American Samoa is in a post-MDA scenario the model is not at endemic equilibrium in 2010. If the reviewer is asking about the initialisation process, the model population and distribution of infections in this population were re-initialised every run, therefore no two runs had identical populations or LF prevalences. We have updated the section on model fitting to state that we used point estimates of parameters for simulations of interventions.

It appears that the implicit reason given here for limiting to 100 runs, though not explicitly stated, is that the authors believe that this will introduce more uncertainty into the results, because there is no extra computational cost to sampling from the posterior. My personal view is I don’t think this is a good reason to throw away the uncertainty – this can give the impression of false precision. I am willing to accept this, though, as now the manuscript is transparent that they are not using the posteriors.

I do note, however, that the manuscript does not say which point estimates are used – is it the MAP, mean or median?

4. *I also have concerns about the low number of replicates used to generate results, which appears to 4 be 100. Given this low number, the results should be presented with an estimate of uncertainty*

Please see response to comment 10 from reviewer 1.

5. *Given the spatial aspect of the study, it might be a nice addition to see maps of prevalence (if possible).*

While it would be possible to generate prevalence maps, we have decided to not include any as this paper focuses on American Samoa as a whole, not predictions for specific locations within American Samoa. Also, due to the limitation of survey data available we were unable to initialise and fit the model with location specific prevalence, instead initialising and fitting to the macroscopic properties of disease distribution (i.e. prevalence of Ag and mf by age group, and ICC at household and village levels). Any predictions of prevalence in specific locations would be quite dependent on initial conditions and have a high degree of random variability over the course of simulation. While prevalence maps from an individual run would be useful for illustrative purposes, it risks misleadingly suggesting that the model is suited for predicting prevalence in specific locations.

If it is not possible due to the nature of the data that is unfortunate but understandable. However, if spatial models are to have utility over simpler models, then it will be in their ability to give spatial predictions, as it is conceivable that a simpler model will be able to fit the aggregated data equally as well, and also give accurate predictions. I think in the discussion it should be stated that making spatial predictions that are accurate is at least an ambition.
